# Contextual Action Theory in Nursing Home Settings: A Conceptual Framework for Considering the Active Role of Residents

**DOI:** 10.1007/s12062-024-09448-7

**Published:** 2024-04-24

**Authors:** Charlotte Jensen, Stephanie Chamberlain, Sheila K. Marshall, Richard A Young, Matthias Hoben, Andrea Gruneir

**Affiliations:** 1 Department of Family Medicine, University of Alberta, Edmonton, AB Canada; 2 Faculty of Nursing, College of Health Sciences, University of Alberta, Edmonton, AB Canada; 3 School of Social Work, Division of Adolescent Health and Medicine, University of British Columbia, Vancouver, BC Canada; 4 Department of Educational and Counselling Psychology, and Special Education, University of British Columbia, Vancouver, BC Canada; 5 School of Health Policy and Management, York University, Toronto, ON Canada

**Keywords:** Nursing home, Quality of care, Resident experience, Personhood, Citizenship, Agency

## Abstract

Nursing home (NH) residents are often considered passive recipients of care with a limited role in shaping their experience. This perspective is often reproduced in NH research, which restricts resident participation, thereby upholding ageist views that cause discrimination of older adults living in NH settings. In this article, we propose using Contextual Action Theory (CAT) as a conceptual framework for exploring NH experiences in a way that incorporates the active role of residents. CAT supports the active role of NH residents by emphasizing the capabilities of human beings to form preferences and act on those preferences, without assumptions of rationality. The emphasis on human action allows researchers to consider NH experiences as co-constructed between residents, care providers, and family members, which means placing an emphasis on the actions and goals of NH residents, no matter their cognitive or physical impairments. CAT also supports personhood and social citizenship concerns in NH research, by acknowledging the differing preferences and thereby differing experiences of NH care by individual residents. We argue that CAT should be considered a useful framework for putting residents in the center of NH research.

## Introduction

NHs provide care to people, who can no longer safely remain in their own homes. Despite the important role that NHs play in the care of this vulnerable population, the reality is that they were designed for and not with older adults. The current state of NHs, and their perceptions, can be directly attributed to ageism (Wyman et al., 2018; McKinley & Adler, 2006; Herron & Aubrecht, 2021). Structural ageism is often a hidden source of inequity that (implicitly and explicitly) introduces bias against older people (Williams et al., 2017). In the case of NHs, structural ageism has manifested as inadequate physical space that is often described as “hospital-like” and leaves residents with limited privacy, insufficient and insufficiently-trained staff to meet residents’ full range of clinical, emotional, and social needs, and division from surrounding communities that can result in residents being isolated from family and friends (Nikmat et al., 2015; Dickens et al., 2011).

The impact of ageism is evident in the design and organization of NH care. Ageism in NHs often manifests as a perception of residents as weak, frail, sick, sad, or angry individuals, who have no preferences and therefore stand on the outside of all social interactions and relationships existing purely as recipients of care itself (Bartlett & O’Connor, 2007; O’Connor et al., 2007; Kitwood, 1997). This ageist view of NH residents can lead to less relationship-centered care, which can negatively impact the residents’ satisfaction with their care (Bowers et al., 2001; Chao & Hsu, 2016). Ageism can also result in an overemphasis on care practices such as task-oriented as opposed to relational care practices that can lead to depersonalization in care staff (Cruz et al., 2020; Gruneir et al., 2023; Halbesleben et al., 2008). Ageism in NH research is also reflected by the often limited inclusion of residents, especially residents with cognitive impairment or other communication disorders, within the research itself (Taylor et al., 2012; Mozley et al., 1999). For many years, research ethics precluded or severely restricted the inclusion of NH residents in research. This was meant to protect vulnerable individuals who may have limited ability to provide consent. While this has changed in recent years, the inclusion of residents with cognitive impairment and their ability to consent and share their experiences continues to be a concern (Sion et al., 2020; Mayo, 2009; Prusaczyk et al., 2017). We argue that excluding residents’ experiences from research on NH care risks reinforcing the ageist view of NH residents as passive, incompetent care recipients unless the agency and capacity of residents to form preferences, experiences, and goals are accurately reflected. To do so we apply contextual action theory (CAT) as a conceptual framework for understanding people’s actions in the context of their goals and relationships with others (Young et al., 2002). It has been used in counseling psychology and social work to understand developmental life transitions but less so for those associated with aging, and to our knowledge, not at all in institutional settings such as nursing homes. This is despite its potential for describing different behaviors and actions in NH residents, who make up a unique but high-interest group in research. In this article, we present a conceptual framework using CAT for researchers to explore resident agency and their role in their own care experience.

We aim to shift the position of NH care research from residents as passive to active participants with agency and goals. The purpose of this article is to describe CAT can work as a useful tool for researchers aiming to involve residents in research about their care in a way that supports their active role in their own care experience.

## Conceptualization of NH Care Research as Joint, Goal-Directed, and Subjective

CAT is a useful conceptual framework for exploring quality of NH care as experienced and reported by residents in a way that supports the capacities and abilities of residents. Below we argue the fit of CAT for addressing issues in researching NH care regarding the capacity, abilities, and goals of NH residents.

### Contextual Action Theory

CAT was originally developed in career and counselling psychology and approaches individuals’ behaviours as embedded in the social contexts, where behaviors are seen as goal-directed and intentional actions. Actions are assumed to reflect socially constructed meanings but not necessarily rational behaviors. The intent of contextual action theory is therefore not to ask ‘why’ people act in certain ways but instead to ask ‘what’ people are doing to understand the socially constructed and underlying meanings that reflect the goals of behaviors (Young et al., 2021). Actions are approached in three different ways: by identifying their systems, their organization and the perspectives that enable us to understand them.

#### Systems of Action

action is considered in systems, which refer to the scale of the action. Actions can be (1) short-term, which typically means a stream of behavior in a moment/interaction that has a specific goal, while being anchored in the daily lives of people related to their cognitive, social, and environmental contexts. When someone constructs several actions with a common goal or meaning within this social/environmental context, we refer to it as (2) projects. Projects happen over a middle-term length of time, which depending on the project can be weeks, months or even years, constructed by several short-term actions. For example, an adult child of a resident of NH might try and encourage their parent to go for daily walks, which is a project that takes place over several weeks or months, made up by daily interactions, walks, conversations, etc., all of which are actions on a short- and middle-term length of time which are driven by a goal of the family staying healthy and active. Depending on how big a part of a persons life these projects are, they can contribute to long-term goals or life goals, which we refer to as (3) careers. Careers represent the construction and organization of projects over a long-term period (often a person’s entire life or a significant portion of that person’s life) with high significance such as wanting to be a good parent, or being a hard worker. (Young et al., 2021; Young et al., 2011).

Actions, whether short-term, projects, or careers, typically involve other people either directly or indirectly, making them joint actions. Joint actions are assumed to serve the goals of multiple actors, and the goals can be similar or divergent. Actions can also be individual, but as people rarely act or exist in a vacuum, most actions indirectly impact, include or are influenced by other people (Young et al., 2021; Young et al., 2011).

#### Organization of Action

actions are also considered on different levels of complexity. (1) Action elements are verbal and nonverbal behavior involved in an action as well as the internal and external resources needed for the execution of the behavior. This could be a statement (verbal behavior) or a head gesture such as a shake or a nod (physical behavior). (2) Functional steps are the means the actor is using to get to the goal, meaning the sequence of linked actions that ultimately will result in the desired end outcome. This could be a series of statements to express an opinion or make a plan. (3) Goals are the final level of complexity and are the end state that actions are directed towards. This also means that goals ascribe the meaning to the actions, both individually but also contextually.

#### Perspectives on Action

Finally, actions are considered with different perspectives on their manifestations including (1) manifest behavior, which refers to observable verbal or nonverbal behavior, (2) internal cognitive and emotional processes, typically thoughts or emotions, that direct the actions, and (3) the social and cultural meanings people construct around behavior, such as a wave being a greeting, to make them actions (Young et al., 2021; Wall et al., 2015).

CAT is especially beneficial for the conceptualization of NH care as joint, goal-directed and subjective because of these different approaches to action. Since actions and goals are the focus, anyone who is able to act on and form preferences is considered an actor and agentic by nature based on CAT (Young et al., 2021; Young et al., 2011). This means that regardless of cognitive abilities, all people are considered to be engaging in goal-directed actions. Previous studies with young children, people with intellectual and learning disabilities as well as hearing impaired people have been conducted and shown how despite physical, developmental, or cognitive restrictions, people have goals, desired end outcomes and they act accordingly (Zaidman-Zait & Young, 2008; Zaidman-Zait et al., 2014; Bartlett & O’Connor, 2007). This offers an approach for researchers to include NH residents with cognitive impairment, who might otherwise face a higher rate of exclusion from research than cognitively unimpaired residents. It also enables researchers to not only look at the provision of NH care or the resident experience as separate elements but rather to consider the active participation of residents as social citizens.

## CAT & Social Citizenship

### The Co-Construction of NH Care

As we outlined in the introduction, NH care is designed for residents but not with them, in an ageist process that has resulted in institutionalized care viewing residents as passive and relatively incompetent recipients of care rather than persons with agency and goals (Wyman et al., 2018). Not only does the design of NH impact how care is provided within the institutional setting, but it also impacts how that care is researched. Most of the tools for documenting quality of care such as the Minimum Data Set (MDS) diminish the focus on resident participation. There is a consistent lack of sustained and meaningful focus on how to create opportunities for co-construction of NH care, which would cement the role and agency of the residents in their own care experience.

The emphasis on action in CAT enables researchers to view NH care as a co-constructed process of individual and joint goal-directed action and shift the way NH care is thought about in the process. NH care typically happens between two people, a care provider who is meeting a need and a resident who has a need that is being met. There is no NH care without someone providing the care to someone who needs the care. Consider the care task of getting a NH resident out of bed. There are NH staff members who are working on the task of getting someone out of bed to further fulfill this person’s need for food, a shower, movement, social interactions, etc. No matter the level of physical participation or cognitive impairment of the resident, the presence of the resident is necessary for the care task to take place. Furthermore, the resident’s need for food, a shower, movement, social interactions, etc. must be present for the immediate care task of getting out of bed to be relevant. The CAT approach enables researchers to consider care as co-constructed and to acknowledge that the meaning and completion of NH care tasks require the presence and the goals of both residents and staff. CAT enables this shift in focus from the care provider acting independently of the resident, to the joint nature of the care task with both the health care provider and the resident equally involved. This consideration of NH care as co-constructed by the actions and goals of residents and care providers is a helpful tool for researchers to avoid reinforcing ageist views of residents as passive and incompetent by excluding their role in the construction of their care.

### Co-Construction of NH Care and Social Citizenship

Considering the residents as active participants in the co-construction of their care also enables support of their personhood and social citizenship. Social citizenship is defined as the justice, recognition of social positions, and the upholding of personhood, rights, and freedom from discrimination of persons with dementia or in this case NH residents with or without cognitive impairment (Bartlett & O’Connor, 2007). Social citizenship is not just seeing the person but considering them as active agents in the social context of their environment. When residents are excluded from research, they are often labeled based on their cognitive impairments and/or diagnoses and the stigma others have of them. This “othering” of the residents based on negative stigma challenges the retention of social citizenship within the context of NH where residents are still active agents with rights, history, competencies and unique preferences, and experiences (O’Connor & Nedlund 2016; O’Connor et al., 2007).

Neglecting how NH residents actively play a role in care activities, not simply by receiving them but by needing, receiving, and reacting to the care, is to ignore the agency that residents have in their day-to-day lives in the NH setting. CAT allows researchers to not only acknowledge residents’ social citizenship in the NH setting but to actively support it by enabling the residents’ capabilities to report on their own experiences of and goals for NH care and more importantly how they act on these goals and preferences. Using CAT to not focus solely on the cognitive impairments or processes surrounding NH care but to focus on the types of actions being taken by different participants is empowering for the residents’ agency.

Related to the social citizenship of the residents, engaging directly with the goals and actions of NH residents can also have a profound effect on the resident’s sense of personhood. The residents do not exist in a vacuum where they simply receive care and have no relationships or interactions. Rather, residents receive care within a socio-cultural context where norms, values, beliefs, and assumptions shape the experiences and how experiences are interpreted and responded to (O’Connor et al., 2007). In these interactions, residents respond to and interpret the messages they receive from others about who they are, which impacts their understanding of themselves and their role. Residents thereby maintain, invent, and reinvent their sense of self through their interactions with others and their environment (Baldwin, 2008). Using CAT to engage with the actions and goals of residents regarding their care supports the existence of a unique and autonomous experience for each resident. Not all residents will have the same goals or preferences for care, and they will therefore not all act the same. Since the goals and actions of residents are a core aspect of the co-construction of NH care, researchers must consider not just the role of residents, but the unique preferences of residents as persons that shape the actions and goals, which makes up NH care. Furthermore, engaging directly with the actions and goals of the residents can support residents’ efforts to maintain a sense of self and thereby supports the residents’ personhood both by acknowledging the fact that they have independent and unique preferences, and by involving them in the unique interactional environment with researchers. CAT enables this approach to individuality, as well as how this manifests in relationships with others, such as care providers.

### NH Care as Goal-Directed Action

As we argued earlier, the actions of one or more people create a sequence of actions that make up NH care. Actions are driven by goals, which can be individual or joint, similar, or divergent. The key is that people act based on their own, individual goals. While NH residents who have cognitive impairments can struggle to report and reflect on their preferences for care, they still act and have goals that drive those actions. Their goals might change, or residents might have a harder time forming courses of action to best achieve their goals, but their actions are still directed by underlying goals. Consider responsive behaviors such as wandering, angry outbursts, etc. While these are often more emotional in nature, they are still driven by cognitively salient goals of changing something, be that their surroundings, their interactions, or the behaviour of others (Young & Domene, 2012). Engaging with resident goals and preferences when conducting NH care research is a valuable way for researchers to steer away from the ageist perspectives of residents’ quality of life being determined entirely by the quality of their care, of which they are considered passive recipients. By instead considering how residents have goals for their care that are unique, even if they have cognitive impairments that hinder the reporting on these goals, researchers enable a perspective on residents as having personal goals driving their actions.

Staff and residents can have convergent goals, such as increasing resident well-being but how they arrive at these shared goals might be different. Staff will provide care through tasks such as personal hygiene, assisting with meals, responding, and reacting with humor, where the resident might engage in activities, spend time with family, or interact with staff. Despite the different ways for staff and residents to achieve their shared goals, they still aim for the same desired outcome. This shared goal creates a joint project between staff and residents of working towards resident well-being through similar or different patterns of actions.

Goals do not, however, have to be convergent to enable a joint project between staff and residents. Staff can want to get paid, get a task done, or consider one aspect of the care task at hand (i.e. administering medication), while the residents’ goals might be to feel good, and have a meaningful interaction as opposing goals to the staff. However, when people have goals, joint or individual, about the same task or interaction that they are both participating in, the action becomes a joint project where both parties are aiming to achieve their unique goals but are unable to do so without the participation of the other.

Residents’ involvement in NH care, therefore, extends beyond experiencing care. Their involvement is determined by their actions, which are directed by their goals. NH care is constructed by the goals and actions of the residents as well as the care partners and the staff. Consider the goal shared between residents and care partners in Gruneir et al. (2023). Here the joint project between the residents and their care partners is to make the time spent in NH as good as possible. This goal, and all the linked actions taken to achieve this (i.e. maintaining relationships and a sense of self, advocacy) requires the agentic nature of residents setting (Gruneir et al., 2023). Another type of project in NH could be between staff and resident, aiming to create social relationships, where staff members might take short-term action to encourage interactions between residents (i.e. encourage mealtime conversation, help start activities between residents, etc.) as part of a middle-term project of supporting social life. Any of these joint projects can include divergent goals (the resident might not want to interact with other residents but might otherwise want to maintain relationships with staff or their care partners), but whether the goals are congruent or not, the actions together create projects.

Careers for residents would be more difficult to determine, as their time spent in NH might not add up to a big enough part of their life (either in terms of number of years spent there or in terms of value or importance to the resident) to be considered as such. One could imagine a staff member making a career of their occupation regarding providing the best care of their ability, which the residents would be a, although passive, part of. However, considering specifically the agentic nature of residents, the relevant systems of action might be limited to projects and actions (middle-term and short-term timelines). It is possible that a resident spending years of their life in NH could make a career of i.e. advocacy for residents, but for this to be relevant, the active participation of the resident in the construction of this career is key. No matter the time frame and the goals, the agency of the resident is core to the construction of NH care as a social process.

Based on a CAT approach, NH care can therefore both be considered co-constructed by the actions of residents and staff, as well as goal-directed based on the preferences that drive the actions of residents and staff. This approach is a beneficial addition to NH care literature as it works against the ageist views of receipts as passive and without an active role in their care. Not only do they have goals and preferences that they act on, but these goals also are essential for the care tasks to take place.

## Discussion

This article aims to outline a conceptual framework for researchers to consider the role of residents in NH care. Throughout this article, we have argued that current NH literature risks reinforcing ageist views of NH residents as passive and incompetent recipients of care. CAT as a conceptual approach enables researchers to consider the agency of residents in (1) how NH care is co-constructed and (2) how NH care is directed by the goals of residents as well as staff. Emphasizing the actions and goals of residents strengthens NH care research by depicting the role of residents in their own care experience.

Two main challenges for conducting this type of NH care research remain that we would like to address. The first is regarding the practicality of this type of framework. NHs are challenging settings to conduct research in and adding data collection from residents can stretch out timelines, and increase the capacity needed from the research team for ethics application, data collection, and data analysis. Second, is the residents’ ability to consent to participate in research. Typically, arguments are made that (1) residents with cognitive impairment might not understand the scope of the research project when presented to them, the meaning of their involvement, or what involvement entails and (2) that consent at one point in time might not mean continued consent as the cognitive impairment worsens or the resident forgets past consent. We argue that consent is not static but is performed within relational contexts (O’Connor et al., 2007). Whoever assesses the ability of the residents to consent should also engage in the processes by explaining, directing/redirecting focus, how the topic is presented, and when it is presented.

An added benefit of CAT as a conceptual approach to NH care research is how it is in line with the concept of relational care. Relational care literature considers a care relationship between caretaker and care receiver to be multi-way and impactful on all participants (not just the recipient) (McCormack et al., 2012; Wilberforce et al., 2017; Koren, 2010). Similarly, CAT creates equal space for the goals and actions of staff as well as residents to make up NH care, which enables perspectives of different care roles. As we argued earlier in this article, CAT also centers on the individuality and agency of residents, which aligns with social citizenship and personhood literature.

CAT enables researchers to view individual behaviors and activities between people as a way for humans to construct and co-construct the self, their relationships, as well as social and cultural aspects of everyday human lives such as NH care. By applying the lens of CAT we can view NH care as a jointly constructed and agentic process that occurs on the individual and group levels. As previously argued, NH residents are often viewed as passive recipients of care both in NH care and in NH care research. To enable both actual and scientific change, we discourage this dichotic way of looking and NH residents as care receivers and NH staff as care providers. NH’s are inherently difficult settings for conducting research, and CAT, and its accompanying action-project method setting (Gruneir et al., 2023; Jensen et al., 2022a, b), needs to be considered carefully for the practical implications for data collection in terms of scheduling, ensuring privacy, entering a space that is simultaneously a home and a place of employment. Particularly the idea of a NH room both being a residents home as well as a place of receiving/giving care is an important element to conducting CAT research physically in the NH’s, but even the conceptual impact of this cannot be overstated. Do residents and care staff exist in a comparable mindset, role or relational attitude towards each other when occupying the same space but with different associations? Research has already been done on the practical application of CAT in NH care research as well as adaptations to data collection that can benefit the setting (Jensen et al., 2022a, b). Despite this past work, we encourage further empirical research to enhance the understanding and role of residents as active agents in their own care experience. Future research can benefit from considering CAT as a theoretical approach to NH care research based on its emphasis on action and goals, which as we outlined in this article, compliments literature on personhood and quality of life.
